# Prevalence and Risk Factors of Problematic Internet Use among Hungarian Adult Recreational Esports Players

**DOI:** 10.3390/ijerph19063204

**Published:** 2022-03-09

**Authors:** Gábor Kósa, Gergely Feher, Lilla Horvath, Ivan Zadori, Zsolt Nemeskeri, Miklos Kovacs, Éva Fejes, Janos Meszaros, Zoltan Banko, Antal Tibold

**Affiliations:** 1 Centre for Occupational Medicine, Medical School, University of Pécs, 7624 Pecs, Hungary; kosagabor@kosagabor.hu (G.K.); lilla.horvath@etk.pte.hu (L.H.); kovacs.miklos.pte@gmail.com (M.K.); meszarosjanosdr@gmail.com (J.M.); tibold.antal@pte.hu (A.T.); 2 Department of Primary Health Care, University of Pécs, 7623 Pecs, Hungary; 3 Faculty of Cultural Sciences, Education and Regional Development, University of Pécs, 7633 Pecs, Hungary; zadori.ivan@pte.hu (I.Z.); nemeskeri.zsolt@pte.hu (Z.N.); 4 Hospital of Komló, 7300 Komlo, Hungary; efchengirl@gmail.com; 5 Department of Labour Law and Social Security Law, Faculty of Law, University of Pécs, 7622 Pecs, Hungary; banko.zoltan@ajk.pte.hu

**Keywords:** esports, internet addiction, epidemiology, demographic, medical condition

## Abstract

Background: Esports are highly prevalent in modern culture, particularly among young people, and are a healthy hobby for the majority of users. However, there is a possible link between video gaming (including esports) and problematic internet use (so-called internet addiction, IA), mostly involving adolescents. Methods: Here we present an online survey focusing on the prevalence and risk factors of internet addiction among adult esports players. Demographics included age, gender, family type, type of work, working years and daily internet use. Medical conditions associated with IA such as smoking, alcohol and drug intake, hypertension, diabetes, ischemic heart disease, musculoskeletal pain and history of depression were also recorded. Results: Overall, 2313 players including 176 females (7.6%) and 2137 males (92.4%) participated in our online survey. Age distribution was the following: 18–25 years 90.3% (2088/2313), 26–35 years 7.95% (184/2313), 36–45 years 0.86% (20/2313), 46–55 years 0.82% (19/2313), 56–62 years 0.04% (1/2313) and 62 years or older 0.04% (1/2313). Internet addiction was detected in 19.9% of players (461/2313) based on the Problematic Internet Use Questionnaire. In a multivariate analysis internet addiction was significantly associated with age between 18 and 25 (OR: 1.675, *p* = 0.002), being single (OR = 1.505, *p* = 0.014), internet use > 6 h daily (OR = 4.338, *p* < 0.001), having < 3 children (OR: 2.037, *p* = 0.023) and having secondary employment (OR = 1.789, *p* = 0.037). Regular alcohol intake (OR = 18.357, *p* < 0.001) and history of depression (OR= 5.361, *p* = 0.032) were also strongly correlated with IA. Conclusion: This is the first study from Hungary investigating the prevalence and risk factors of internet addiction among adult esports players. One out of five adult gamers suffered from IA. Our study also draws attention to increased risk within this group and risk factors such as younger age, family status and type of employment.

## 1. Introduction

Problematic internet use (PIU) or internet addiction (IA) is one of the most extensively studied phenomena of our century, generating debates among experts and researchers [1]. The key issue is prolonged, compulsive and problematic use with significant impairment in various domains of life, similar to other types of conventional addictions such as alcohol misuse [2]. PIU is not a single diagnosis; it must be regarded as an umbrella term covering all aspects of compulsive consumption such as social media addiction or gaming disorder. Problematic users do not have the ability to control their online activities, which adversely affects their private and social lives and vocational achievement, leading to lower quality of life [3]. 

Despite three decades of intensive research on the topic, the classification of IA (including diagnostic methods, symptomatology and treatment) is still incomplete. Although its symptomatology suggests that it should be regarded as a compulsive–impulsive spectrum disorder, IA was not included in the latest (5th) edition of the Diagnostic and Statistical Manual (DSM-V) [4,5]. 

Its prevalence has been increasing over time, and based on a recent meta-analysis it may affect about 7% of the whole population, it is highly prevalent among adolescents (potentially over 20%, but geographical differences occur) and there is a decreasing tendency with age [6].

Male gender, early internet use, low family income and metropolitan life carry a higher risk of problematic internet use, as do several personality traits such as higher impulsivity, sensation seeking and lower self-directedness [7,8]. 

Family functioning can have a crucial role, as poor intra-familiar relationships and inappropriate parental support or monitoring are strongly associated with IA, especially in cases of early exposure to the internet [9]. 

Inability to stop online activities and spending increasing amounts of time online, as well as bedtime internet use, are indicators of tolerance. Daily amount of time spent online seems to be strongly associated with IA, and several activities such as gaming (especially for males) and social media use (especially for females) carry a higher risk of dependence [4,7]. 

Although there is a strong link between problematic internet use and a wide range of mental issues such as anxiety/depression, substance abuse, poor sleep quality/insomnia and attention deficit hyperactivity disorder (ADHD), the association has not entirely been clarified as only cross-sectional studies exist [10,11,12,13,14]. However, recent studies have shown common vulnerability factors and bidirectional interactions between ADHD and gaming disorder, raising the possibility of the need for screening for this condition among problematic gamers and internet addicts [15,16]. Furthermore, depression and hopelessness were also found to be more common among gaming addicts in [17].

Although internet addiction is not labelled as a medical condition, gaming disorder was included both in the appendix of the DSM-V (as a potential warning condition) and in the ICD-11 as a medical condition [18,19]. 

Video gaming (or esports) is a common name for competitive online video gaming, which is extremely prevalent in our century and is considered a healthy hobby for the vast majority of people (also promoted as such by developers) [20]. Conventional sports and esports (competitive online video gaming) share similarities such as registered players, the need for regular training (including motor skills, hand–eye coordination, problem solving and reaction time), semi-professional or professional teams with managers, (online) leagues with competitions, championships and prize money [21]. Video gaming or esports has become a multibillion-dollar business involving tens of millions of players and an audience of hundreds of millions [20,21]. The million-dollar competition prizes lure amateurs to pursue it professionally, but only a minority of them can build a career in esports; a significant percentage of participants cannot make their living as players, as they are employed elsewhere in part- or full-time jobs [22,23]. 

It seems reasonable to expect an increase in problematic gaming among esports players, as recent studies have shown that top gamers may play up to 22 h a week; however, only a few papers have been published in the academic sector, medical community or health-related disciplines on the potential adverse impacts and effects of esports, as summarized by a recent systematic review [23]. Adolescents interested in video gaming (so-called recreational players or semi-professional gamers, which make up the vast majority of players) may spend more than 5 h a day playing in addition to their daily activities [20,21,22,23,24]. Problematic gaming affects only a minority of players, but due to the large number of participants it may have a significant impact on healthcare. Prolonged gaming and internet use can be associated with physical symptoms such as eye strain, blurry vision, musculoskeletal pain and headache [21,23]. Mental issues can include mood disorders (depression, anxiety, apathy, emotional disturbances), poor sleep quality, aggressive behavior (probably due to violent games) and social isolation [21,23]. 

More than 90% of these players are male, and regular players (both professional and recreational users) spend their days with excessive online gaming or watching online matches as mentioned above (it is important to remember that both male gender and gaming as the purpose of internet use are strongly associated with problematic internet use) [25]. 

It is worth mentioning that daily recreational computer use (outside esports) including watching video tournaments or surfing on social sites can reach more than 4 h among gamers, according to the findings of a recent study [26]. Esports players are likely to spend 6–8 h or more a day gaming and with other types of internet use.

Very little (and low quality) data are available on the effect of esports on problematic gaming or internet gaming disorder, and there are no studies focused on the possible effect of esports on IA. The above-mentioned studies focused on adolescents and problematic gaming, but no or limited data exist for adult populations (regarding problematic gaming) and there is a lack of data on IA in both populations.

Therefore, we aimed to carry out an online cross-sectional survey to (1) detect the rate of internet addiction among adult esports players (playing competitive online video games, no digitalized sports or similar physical activities); (2) include demographic parameters such gender, age, family type, number of children, education and type of employment including weekly working hours, as well as daily internet use; (3) and collect information on risk factors and medical conditions such as smoking, alcohol and drug consumption, diabetes, hypertension, cardiovascular disease, musculoskeletal pain and history of depression. 

## 2. Materials and Methods

### 2.1. Participants

This cross-sectional study was conducted between March and August 2020. The study was approved by the Ethics Committee of the University of Pécs (8434-PTE 2020). Study participants were recruited online, coordinated by Universum 8 Zrt via twitch.com with the help of two influencers (see Acknowledgements). A detailed online survey was conducted (see above). Only gamers aged > 18 years and who signed the consent form prior to completing the survey were allowed to participate. Participation was anonymous and voluntary. 

### 2.2. Data Collection Instrument

As there are no clear diagnostic criteria for internet addiction, it is highly recommended that excessive internet use is measured with a continuous questionnaire [27]. We chose the Problematic Internet Use Questionnaire (PIUQ) because its structure tightly adheres to the proposed diagnostic criteria for internet addiction and was created based on clinimetric and psychometric analysis of Young’s Internet Addiction Test, independently validated by several groups and used in our previous published work [2,28,29,30,31,32]. The questionnaire contained 18 items, each scored on a 5-point Likert-type scale ranging from 1 (never) to 5 (always). A confirmatory factor analysis verified the three-factor model of the questionnaire, with each subscale containing six items. The obsession subscale refers to obsessive thinking about the internet (daydreaming, rumination and fantasizing) and withdrawal symptoms caused by the lack of internet use (anxiety and depression)—“How often do you feel tense, irritated, or stressed if you cannot use the internet for as long as you want to?”. The neglect subscale contains items about neglecting everyday activities, social life, and essential needs (“How often do you spend time online when you’d rather sleep?”). The control disorder subscale reflects difficulties in controlling time spent on the internet (“How often do you realize saying when you are online, “just a couple of more minutes and I will stop”?”). Since in this study we focused on global psychological consequences of internet addiction, we used the PIUQ total score in the statistical analyses, which was computed by summing the scores on all the items of the scale. A total score exceeding 41 points suggests internet addiction [33].

### 2.3. Process and Data Analysis

Based on the PIUQ results, participants were categorized as (1) problematic internet users (internet addiction) or (2) normal users (not addicted to the internet). Data were evaluated as mean ± SD (standard deviation) by Student’s *t*-test or chi-square test to detect significant differences among examined parameters. To clarify the role of different parameters as independent risk factors for problematic internet use, logistic regression analysis was carried out including all the examined parameters (demographic data, risk factors, concomitant diseases and internet use). For all odds ratios, an exact confidence interval (CI) of 95% was constructed. Data analysis was performed using SPSS (version 22.0, IBM, New York, NY, USA).

## 3. Results

### 3.1. Demographics

Demographics included gender, age, family type, number of children, education and type of employment, including weekly working hours.

A total of 2313 esports players completed the online questionnaire, of which 92.4% were males. Age distribution was as follows: 18–25 years 90.3% (2088/2313), 26–35 years 7.95% (184/2313), 36–45 years 0.86% (20/2313), 46–55 years 0.82% (19/2313), 56–62 years 0.04% (1/2313) and 62 years or older 0.04% (1/2313). 

The vast majority of our study population were single and had no children. More than 90% had elementary school or secondary education and fewer than 10% had a higher education degree. 

About 70% had any kind of employment and the others were still studying. More than 50% had a regular work schedule (full-time or part-time) and about 20% had a secondary employment. More than half of our study population worked 20 h or more in a week (Table 1).

### 3.2. Risk Factors and Previous Diseases

Risk factors included smoking, alcohol and drug consumption habits (relatively regularly or not). Previous diseases considered were history of diabetes, hypertension, cardiovascular disease, musculoskeletal pain and depression.

Among the study population, 25.3% were regular smokers, 14.1% consumed alcohol and 22.0% were using illicit drugs more or less regularly, while 11.6% were regularly taking medication. 

Among those studied, 8.5% had hypertension, 2.3% had diabetes and 3.6% had ischemic heart disease. Furthermore, 2.6% suffered from musculoskeletal pain and 2.9% had a history of depression (Table 2).

### 3.3. Internet Use

Daily internet use was also recorded; 2.5% spent 1 h, 13.9 spent 2 h, 21.8 spent 3 h, 22.6% spent 4 h, 16.2% spent 5 h and 15.7% used the internet more than 6 h a day (not shown).

### 3.4. Prevalence and Risk Factors of Internet Addiction 

Internet addiction was detected in 19.9% of the study population, based on the Problematic Internet Use Questionnaire. Internet addiction was more common in workers aged between 18 and 25 (*p* = 0.029). IA was more prevalent among singles (*p* = 0.020) and those with children (*p* < 0.05) (Table 3). Being a student (*p* = 0.03) carried a higher risk of problematic internet use, as did having casual (*p* = 0.03) or secondary employment (*p* = 0.036). Spending less than 10 h a week with work was also significantly associated with IA (*p* = 0.035) (Table 3). 

Internet addiction was significantly prevalent among alcohol users (*p* = 0.002), in hypertensive individuals (*p* = 0.0018) and among gamers with history of depression (*p* < 0.01).

There was a significant association between the time spent online and being addicted to the internet (r = 0.020, r^2^ = 0.143, *p* < 0.001). The cut-off value predicting IA was spending 6 h or more online (Table 4).

In a multivariate analysis including all factors (demographic data, risk factors, concomitant diseases and daily internet use) internet addiction was significantly associated with age between 18 and 25 (OR = 1.675, *p* = 0.002), being single (OR = 1.505, *p* = 0.014), surfing the internet ≥6 h daily (OR = 4.338, *p* < 0.001), having children (OR = 2.037, *p* = 0.023) and having secondary employment (OR = 1.789, *p* = 0.037). Regular alcohol intake (OR = 18.357, *p* < 0.001) and history of depression (OR = 5.361, *p* = 0.032), were also strongly correlated with IA (Table S1). 

## 4. Discussion

Esports are a billion-dollar industry in the 21st century, attracting millions of players and viewers. Recent publications have shown a possible link between esports and problematic (compulsive) online gaming, but no publications have focused on their association with IA [20,22]. As gamers are likely to spend as much time gaming as they spend online (as recreational activity), we hypothesized that esports are strongly related to problematic internet use—a connection that has not been investigated in any study published to date.

Our findings revealed a rate of internet addiction of nearly 20%, which is significantly higher than the previously described 7% average and much higher than our earlier data on other adult populations [6,32,34]. Based on our previous results, about 5% of adults can be affected with IA; therefore, online gaming (esports) could be strongly linked to problematic internet use, which merits further investigation [34]. 

Younger age was found in both univariate and multivariate analysis to be strongly associated with IA, which is consistent with previous data highlighting the importance of younger age as a potential risk factor [35,36].

More than 90% of our study population consisted of males, similar to the results of previous studies among esports (competitive video gaming) players. There is no clear explanation for this phenomenon. It may be attributable to gaming not being considered by women as a career option, a possible fear of sexism or to differences in socialization. Furthermore, women are usually not a target group of the gaming industry. However, these suggestions are merely speculative and are based on online comments and interviews; they should therefore should be considered carefully [37]. Interestingly, male gender was not a predictor of IA, which is inconsistent with previous results [32,34]. 

Being single, living as a student and working <10 h per week were associated with IA in a univariate analysis. This may be attributable to the above parameters predisposing individuals to having more free time, and the fact that leisure boredom may enhance online gaming and other activities which can lead to addiction [38]. On the other hand, only being single was a significant predictor of IA in a multivariate analysis. Apart from leisure boredom, turning to online gaming can be a coping strategy for loneliness or deficient social skills among affected people [25,35]. 

Having children, non-clarified employment or secondary employment also carried a higher risk of IA. In our previous papers, having a larger family and being overwhelmed with work were stronger predictors of problematic internet use [2,32,34]. In these populations it is possibly escapism (from real-life problems) that could be the main motivation for turning to and then being absorbed by the internet [25,35]. Having children and secondary employment also proved to be independent predictors of problematic internet use. 

Only approximately 3% of our study population spent <1 h online daily, the vast majority spent >3 h on online activities and about 20% reached daily internet use of 6 ≥ hours. There was a significant correlation between being online and IA, in line with previous studies [32,34]. Spending more than 2 h online daily was suggested as the cut-off value for addiction based on previous studies. Nonetheless, similar to our results in adult Hungarian populations, the present study showed the six-hour time limit to be an independent predictor of IA [9,32,34].

IA was also associated with alcohol intake and previous history of mood disorder (depression). The association between depression and IA is well-documented, but causality is not entirely clarified [2,39]. A pre-existing mood disorder may enhance internet use as a coping mechanism; conversely, problematic internet use may lead to subsequent depression. As a third possibility, they may precipitate each other [39]. Similar association (with a similar explanation) was found between substance abuse and problematic internet use [40]. The above parameters were significantly associated with IA in a multivariate analysis, further emphasizing their clinical importance. 

Hypertension was associated with IA among our study participants, which has rarely been documented. Spending many hours online can be associated with a sedentary lifestyle and obesity, which can lead to hypertension [41]. Furthermore, being inactive can result in high body fat mass and low lean body mass, which may predispose individuals to metabolic syndrome and diabetes, conditions that are strongly associated with hypertension [42]. A recent study showed that esports players had more sedentary/inactive lifestyles and higher body-fat percentage and lower lean body mass despite a normal body mass index, compared to age-matched controls [26]. Nonetheless, hypertension was not significantly associated with problematic internet use in a multivariate analysis. 

In summary, this is the first study in Hungary and worldwide focusing on the prevalence and risk factors of internet addiction among recreational adult esports players. IA is very common in this population, suggesting a strong relationship. Our study also draws attention to the risk factors for IA, such as younger age, family status and type of employment, and its possible association with depression and alcohol use (abuse). 

However, our results should be interpreted carefully. Further studies are needed to establish the diagnostic criteria of internet addiction as well as its possible treatment and long-term consequences. Internet addiction is better considered an umbrella term than a single diagnosis, and it is still a matter of debate whether problematic gaming or gaming disorder is a part of IA or a different entity (similar to social media addiction, online gambling, online porn addiction etc.). It is not entirely clear whether the increased rate of IA originates from problematic gaming among our study participants or has a different etiology. Nevertheless, esports bring an increased risk of technology addiction, which should be kept in mind.

Finally, our article has some limitations. Although this was a cross-sectional study including more than 2100 recreational players, it was not representative of internet addiction in the general/adult population. We also considered depression as the most common mental illness, but we did not take into account ADHD or similar conditions, which can be strongly related to IA. As it was a questionnaire-based online survey, physical examination was not carried out and we had no detailed information about the medical history of the study population, such as type and duration of medical conditions. The above-mentioned limitations may have influenced our findings. Additionally, there was no follow-up carried out.

## Figures and Tables

**Table 1:** Characteristics of the study population.

(*N* = 2313)	%
Gender	
Female	7.6 (176)
Male	92.4 (2137)
Age	
18–25 years	90.3 (2088)
26–35 years	7.95 (184)
36–45 years	0.86 (20)
46–55 years	0.82 (19)
56–62 years	0.04 (1)
above 62 years	0.04 (1)
Marital status	
single	71.0 (1643)
in a relationship	25.9 (600)
married	2.6 (59)
divorced/widowed	0.5 (11)
Number of children	
no children	97.2 (2249)
1 child	1.3 (30)
2 children	1.1 (25)
more than 3 children	0.4 (9)
Education background	
elementary education	30.1 (696)
secondary education	60.0 (1388)
higher education	9.9 (229)
Employment status	
employment	34.8 (804)
entrepreneur	3.5 (82)
student	30.1 (696)
other	31.6 (731)
Work schedule	
full time	35.4 (819)
part time	14.1 (326)
flexible	13.5 (313)
other	37.0 (855)
Time spent with work	
less than 10 h	36.4 (842)
10–20 h	13.3 (308)
20–30 h	9.7 (225)
30–40 h	18.1 (419)
more than 40 h	22.4 (519)
Secondary employment	
no	79.7 (1843)
yes	20.3 (470)

**Table 2:** Previous conditions and diseases in the study population.

Concomitant Diseases (%)	
on regular medication	11.6 (268/2313)
smoking	25.3 (586/2313)
alcohol use	14.1 (325/2313)
drug use	22.0 (508/2313)
diabetes	2.3 (54/2313)
hypertension	8.5 (197/2313)
cardiovascular disease	3.6 (83/2313)
musculoskeletal pain	2.6 (59/2313)
history of depression	2.9 (68/2313)

**Table 3:** Comparison of baseline characteristics of the study subgroups (* *p* < 0.05).

	Not Addicted to Internet (*n* = 1852)	Internet Addiction (*n* = 461)
Gender		
Male	92.7% (1717/1852)	91.1% (420/461)
Female	7.3% (135/1852)	8.9% (41/461)
Age		
18–25 years	89.5% (1658/1852)	93.3% (430/461) *
26–35 years	8.4% (155/1852)	6.3% (29/461)
36–45 years	0.9% (18/1852)	0.4% (2/461)
46–55 years	1.0% (19/1852)	0% (0/461)
56–62 years	0.1% (1/1852)	0% (0/461)
above 62 years	0.1% (1/1852)	0% (0/461)
Marital status		
single	70.3% (1302/1852)	74.0% (341/461) *
in a relationship	26.3% (488/1852)	24.3% (112/461)
married	3.0% (52/1852)	1.5% (7/461)
divorced/widowed	0.4% (8/1852)	0.2% (1/461)
Number of children		
no children	96.8% (1792/1852)	99.1% (457/461)
1 child	1.5% (28/1852)	4.5% (2/461) *
2 children	1.2% (23/1852)	4.5% (2/461) *
more than 3 children	0.5% (9/1852)	0% (0/461)
Education background		
elementary education	29.4% (545/1852)	32.8% (151/461)
secondary education	60.6% (1122/1852)	57.7% (266/461)
higher education	10.% (185/1852)	9.5% (44/461)
Employment status		
employment	36.8% (682/1852)	26.5% (122/461)
entrepreneur	3.6% (67/1852)	3.3% (15/461)
student	29.1% (539/1852)	34.1% (157/461) *
other	30.5% (564/1852)	36.1% (167/461) *
Work schedule		
full time	37.0% (686/1852)	28.8% (133/461)
part time	13.7% (253/1852)	15.8% (73/461)
flexible	13.3% (246/1852)	14.5% (67/461)
other	36.0% (667/1852)	40.9% (188/461)
Time spent with work		
less than 10 h	35.4% (655/1852)	40.6% (187/461) *
10–20 h	13.0% (240/1852)	14.8% (68/461)
20–30 h	9.9% (183/1852)	9.1% (42/461)
30–40 h	18.6% (344/1852)	16.3% (75/461)
more than 40 h	23.1% (430/1852)	19.2% (89/461)
Secondary employment		
no	80.7% (1494/1852)	75.7% (349/461)
yes	19.3% (358/1852)	24.3% (112/461) *

**Table 4:** Comparison of concomitant diseases, substance abuse and daily internet use in the study subgroups (* *p* < 0.05, ** *p* < 0.001).

	Not Addicted to Internet (*n* = 1852)	Internet Addiction (*n* = 461)
Concomitant diseases		
medication use	11.4% (212/1852)	12.1% (56/461)
current smoker	26.1% (484/1852)	22.1% (102/461)
alcohol use	12.8% (237/1852)	19.1% (88/461) *
drug use	21.7% (402/1852)	22.9% (106/461)
diabetes	2.2% (40/1852)	3.0% (14/461)
hypertension	7.5% (138/1852)	12.8% (59/461) *
cardiovascular disease	3.8% (70/1852)	4.9% (23/461)
musculoskeletal pain	2.4% (44/1852)	3.2% (15/461)
history of depression	2.3% (43/1852)	5.4% (25/461) **
Daily internet use (approximately)		
1 h	2.8% (52/1852)	0.9% (6/461)
2 h	15.1% (279/1852)	9.3% (43/461)
3 h	23.2% (430/1852)	16.1% (74/461)
4 h	22.7% (421/1852)	22.1% (102/461)
5 h	15.6% (289/1852)	18.4% (85/461)
6 h	6.7% (124/1852)	9.5% (44/461) *
>6 h	13.9% (260/1852)	23.2% (107/461) **

## Data Availability

The dataset supporting the conclusions of this article is available on request to the corresponding author.

## Supplementary Materials

The following supporting information can be downloaded at: https://www.mdpi.com/article/10.3390/ijerph19063204/s1, Table S1: Results of the multivariate analysis.
