# Supplementary material for: Prevalence and Risk Factors of Problematic Internet Use among Hungarian Adult Recreational Esports Players
Source: Int J Environ Res Public Health. 2022 Mar 9;19(6):3204. doi: 10.3390/ijerph19063204 (PMC8949934; doi:10.3390/ijerph19063204)
Supplement: Supplementary file 1 [file ijerph-19-03204-s001.zip › ijerph-1621451-supplementary.pdf]

Table S1. Results of the multivariate analysis.

	**OR**	***p***
Gender		
Male	0.347	0.566
Female	0.873	0.095
Age (years)		
18–25 years	1.675	0.002
26–35 years	1.039	0.354
36–45 years	0.681	0.404
46–55 years	1.795	0.496
56–62 years	1.595	0.111
more than 62 years	1.511	0.073
Marital status (%)		
single	1.505	0.014
relationship	0.927	0.517
married	1.774	0.706
divorced / widow	0.140	0.927
Number of children		
have no child	1.174	0.150
1 child	2.089	0.023
2 children	2.037	0.020
more than 3 children	0.424	0.388
Graduation		
elementary	2.746	0.064
secondary education	1.118	0.905
higher education	1.298	0.195
Years spent with studying		
no longer studyíng	5.084	0.278
1–12 months	1.762	0.143
12–24 months	2.138	0.696
14–36 months	2.282	0.120
36–48 months	1.973	0.669
6–10 years	0.936	0.150
11–15 years	0.859	0.080
more than 15 years	0.870	0.550
Employment status		
employment	0.398	0.742
entrepreneur	0.549	0.945
student	1.252	0.760
other	0.569	0.070
Work shedule		
full time	0.369	0.885
part time	0.527	0.150
flexibility	0.702	0.906
other	0.712	0.464
Time spent with work		
less than 10 hours	0.602	0.061
10–20 hours	0.776	0.054
20–30 hours	0.861	0.845
30–40 hours	0.708	0.306

more than 40 hours	0.474	0.140
Secondary employment		
no	0.497	0.675
yes	1.789	0.037
Concomittant diseases		
taking any medication regularly	1.009	0.315
smoker	9.098	0.003
taking alcohol	18.537	0.000
taking drugs	1.384	0.240
diabetes	0.573	0.318
hypertension	0.767	0.088
cardiovascular disease	1.541	0.215
musculosceletal pain	0.881	0.267
history of cancer	1.481	0.062
history of depression	5.361	0.001
Daily internet use (approximately)		
1 hour	1.497	0.427
2 hours	0.810	0.420
3 hours	0.728	0.852
4 hours	0.723	0.260
5 hours	0.779	0.655
6 hours	1.987	0.053
>6 hours	4.338	0.001
